# 2,2′-[2,5-Bis(hex­yloxy)-1,4-phenyl­ene]dithio­phene

**DOI:** 10.1107/S160053681202404X

**Published:** 2012-06-02

**Authors:** Chin Hoong Teh, Muhammad Mat Salleh, Mohamed Ibrahim Mohamed Tahir, Rusli Daik, Mohammad B. Kassim

**Affiliations:** a School of Chemical Sciences & Food Technology, Faculty of Science & Technology, Universiti Kebangsaan Malaysia, 43600 Bangi, Selangor, Malaysia; b Institut of Microengineering and Nanoelectronics (IMEN), Universiti Kebangsaan Malaysia, UKM 43600 Bangi, Selangor, Malaysia; c Department of Chemistry, Faculty of Science, Universiti Putra Malaysia, 43400 UPM Serdang, Selangor, Malaysia

## Abstract

The asymmetric unit of the title compound, C_26_H_34_O_2_S_2_, comprises one half-mol­ecule located on an inversion centre. The thio­phene groups are twisted relative to the benzene ring, making a dihedral angle of 5.30 (7)°, and the *n*-hexyl groups are in a fully extended conformation. In the crystal, there are short C—H⋯π contacts involving the thio­phene groups.

## Related literature

For the synthesis and general background references, see: Carle *et al.* (2010 ▶); Promarak & Ruchirawat (2007 ▶); Bouachrine *et al.* (2002 ▶).
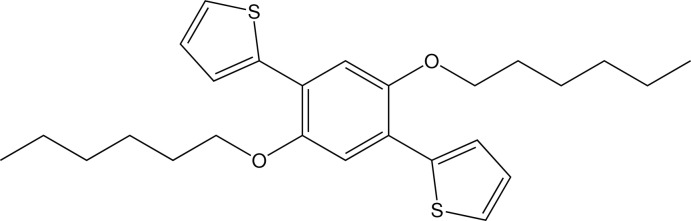


## Experimental

### 

#### Crystal data

C_26_H_34_O_2_S_2_
*M* *_r_* = 442.65
Monoclinic, 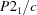

*a* = 12.2996 (3) Å
*b* = 5.4298 (1) Å
*c* = 17.6872 (4) Å
β = 103.982 (2)°
*V* = 1146.23 (4) Å^3^
*Z* = 2
Cu *K*α radiation
μ = 2.25 mm^−1^
*T* = 150 K
0.26 × 0.11 × 0.03 mm

#### Data collection

Oxford Diffraction Gemini diffractometer
Absorption correction: multi-scan (*CrysAlis PRO*; Oxford Diffraction, 2006 ▶) *T*_min_ = 0.592, *T*_max_ = 0.935
8010 measured reflections
2216 independent reflections
2018 reflections with *I* > 2σ(*I*)
*R*_int_ = 0.028

#### Refinement

*R*[*F* ^2^ > 2σ(*F* ^2^)] = 0.040
*wR*(*F* ^2^) = 0.113
*S* = 1.04
2216 reflections
137 parameters
H-atom parameters constrained
Δρ_max_ = 0.40 e Å^−3^
Δρ_min_ = −0.22 e Å^−3^

### 

Data collection: *CrysAlis CCD* (Oxford Diffraction, 2006 ▶); cell refinement: *CrysAlis CCD*; data reduction: *CrysAlis RED* (Oxford Diffraction, 2006 ▶); program(s) used to solve structure: *SHELXS97* (Sheldrick, 2008 ▶); program(s) used to refine structure: *SHELXL97* (Sheldrick, 2008 ▶); molecular graphics: *SHELXTL* (Sheldrick, 2008 ▶); software used to prepare material for publication: *SHELXTL*, *PLATON* (Spek, 2009 ▶) and *publCIF* (Westrip, 2010 ▶).

## Supplementary Material

Crystal structure: contains datablock(s) I, global. DOI: 10.1107/S160053681202404X/gk2496sup1.cif

Structure factors: contains datablock(s) I. DOI: 10.1107/S160053681202404X/gk2496Isup2.hkl

Supplementary material file. DOI: 10.1107/S160053681202404X/gk2496Isup3.cml

Additional supplementary materials:  crystallographic information; 3D view; checkCIF report

## Figures and Tables

**Table 1:** Hydrogen-bond geometry (Å, °) *Cg*1 is the centroid of the S1, C4–C7 ring.

*D*—H⋯*A*	*D*—H	H⋯*A*	*D*⋯*A*	*D*—H⋯*A*
C5—H5⋯*Cg*1^i^	0.93	2.85	3.5809 (16)	137

Symmetry code: (i) 
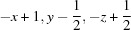
.

**Fig. 1.**
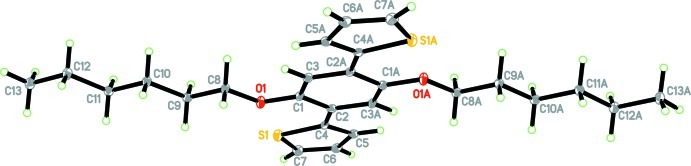
The molecular structure of the title compound, with displacement ellipsoids drawn at the 30% probability level. Symmetry code for atoms with the A label: -x, 1 - y, 1 - z.

**Fig. 2.**
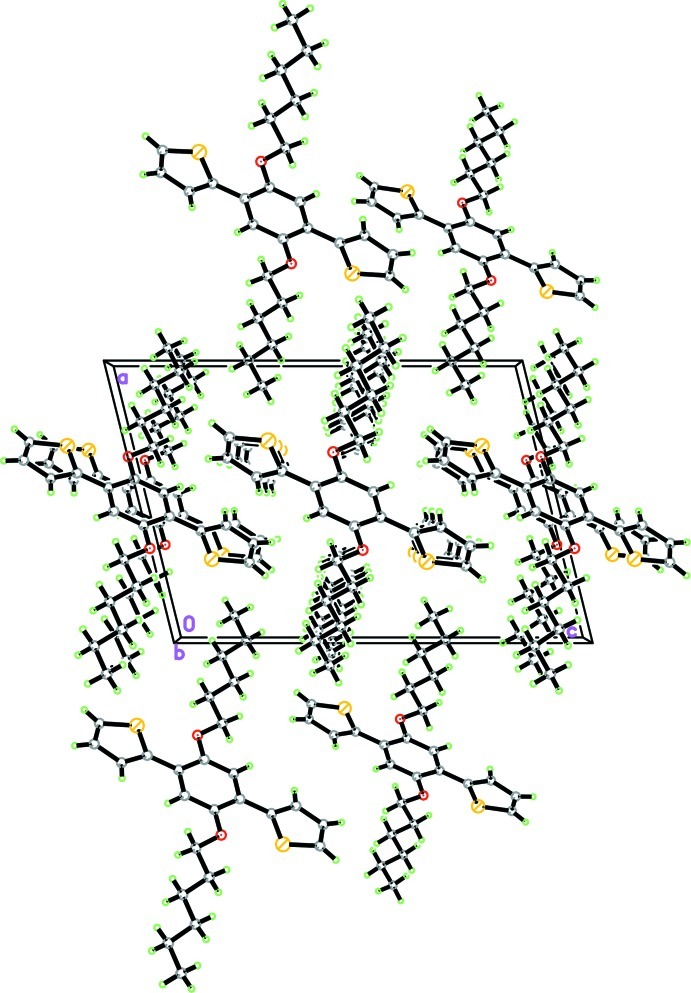
Crystal packing of the title compound viewed down the b-axis.

## supplementary crystallographic information

### Comment

Thiophene-phenylene-thiophene unit, as in the title compound, is an interesting material to produce soluble electroluminescent materials for LED applications (Bouachrine *et al.*, 2002) and making photovoltaic devices (Carle *et al.*, 2010). The solubility characteristic for the title compound in organic solvents was enhanced by the presence of dialkyloxy groups on the phenylene fragment.

The molecule of the title compound is shown in Fig. 1 and crystal packing projection along the *b* axis is shown in Fig. 2.

### Experimental

The preparation of title compound was adapted from previously published procedure with a slight modification (Promarak & Ruchirawat, 2007). Aqueous sodium carbonate solution (2*M*, 10.5 ml) was added into a solution of 2,5-dibromo-1,4-bis(hexyloxy)benzene (1.50 g, 3.44 mmol) in dry THF prior to addition of Pd(PPh_3_)_4_ (0.21 g) catalyst. This was followed by the addition of 2-thiophene boronic acid (1.32 g, 10.32 mmol) and the mixture was heated under reflux overnight in dry N_2_ atmosphere and allowed to cool to ambient temperature prior to addition of water. The product was extracted into CH_2_Cl_2_ and the organic phase was combined, washed with water and brine solution, followed by drying over anhydrous MgSO_4_. The solvent was evaporated using rotary evaporator and the product was further recrystallized from ethanol/ethyl acetate to afford crystals suitable for single-crystal X-ray diffraction (yield: 80%).

### Refinement

The H atom positions were calculated geometrically and refined in a riding model approximation with C–H bond lengths in the range 0.93–0.97 Å and *U*_iso_(H) = 1.2*U*_eq_(C) except methyl group where *U*_iso_(H) = 1.5*U*_eq_(C).

### Crystal data

**Table:**

C_26_H_34_O_2_S_2_	*F*(000) = 476
*M**_r_* = 442.65	*D*_x_ = 1.283 Mg m^−^^3^
Monoclinic, *P*2_1_/*c*	Melting point = 369–367 K
Hall symbol: -P 2ybc	Cu *K*α radiation, λ = 1.54178 Å
*a* = 12.2996 (3) Å	Cell parameters from 3985 reflections
*b* = 5.4298 (1) Å	θ = 4–71°
*c* = 17.6872 (4) Å	µ = 2.25 mm^−^^1^
β = 103.982 (2)°	*T* = 150 K
*V* = 1146.23 (4) Å^3^	Thin plate, colourless
*Z* = 2	0.26 × 0.11 × 0.03 mm

### Data collection

**Table:**

Oxford Diffraction Gemini diffractometer	2216 independent reflections
Radiation source: fine-focus sealed tube	2018 reflections with *I* > 2σ(*I*)
Graphite monochromator	*R*_int_ = 0.028
ω scans	θ_max_ = 71.3°, θ_min_ = 3.7°
Absorption correction: multi-scan (*CrysAlis PRO*; Oxford Diffraction, 2006)	*h* = −15→14
*T*_min_ = 0.592, *T*_max_ = 0.935	*k* = −6→6
8010 measured reflections	*l* = −21→15

### Refinement

**Table:**

Refinement on *F*^2^	Primary atom site location: structure-invariant direct methods
Least-squares matrix: full	Secondary atom site location: difference Fourier map
*R*[*F*^2^ > 2σ(*F*^2^)] = 0.040	Hydrogen site location: inferred from neighbouring sites
*wR*(*F*^2^) = 0.113	H-atom parameters constrained
*S* = 1.04	*w* = 1/[σ^2^(*F*_o_^2^) + (0.0739*P*)^2^ + 0.5049*P*] where *P* = (*F*_o_^2^ + 2*F*_c_^2^)/3
2216 reflections	(Δ/σ)_max_ < 0.001
137 parameters	Δρ_max_ = 0.40 e Å^−^^3^
0 restraints	Δρ_min_ = −0.22 e Å^−^^3^

### Special details

**Table:**

Experimental. The crystal was placed in the cold stream of an Oxford Cryosystems open-flow nitrogen cryostat (Cosier & Glazer 1986) with a nominal stability of 0.1 K.Cosier, J. & Glazer, A.*M*., (1986)., *J. Appl. Cryst.*105 107.
Geometry. All e.s.d.'s (except the e.s.d. in the dihedral angle between two l.s. planes) are estimated using the full covariance matrix. The cell e.s.d.'s are taken into account individually in the estimation of e.s.d.'s in distances, angles and torsion angles; correlations between e.s.d.'s in cell parameters are only used when they are defined by crystal symmetry. An approximate (isotropic) treatment of cell e.s.d.'s is used for estimating e.s.d.'s involving l.s. planes.
Refinement. Refinement of *F*^2^ against ALL reflections. The weighted *R*-factor *wR* and goodness of fit *S* are based on *F*^2^, conventional *R*-factors *R* are based on *F*, with *F* set to zero for negative *F*^2^. The threshold expression of *F*^2^ > σ(*F*^2^) is used only for calculating *R*-factors(gt) *etc*. and is not relevant to the choice of reflections for refinement. *R*-factors based on *F*^2^ are statistically about twice as large as those based on *F*, and *R*- factors based on ALL data will be even larger.

### Fractional atomic coordinates and isotropic or equivalent isotropic displacement parameters (Å^2^)

**Table:**

	*x*	*y*	*z*	*U*_iso_*/*U*_eq_	
S1	0.29977 (3)	0.23308 (7)	0.14406 (2)	0.02015 (17)	
O1	0.33781 (9)	0.3460 (2)	0.00566 (6)	0.0185 (3)	
C1	0.41842 (12)	0.1767 (3)	0.00089 (8)	0.0151 (3)	
C2	0.44944 (12)	0.0140 (3)	0.06456 (8)	0.0146 (3)	
C3	0.46770 (12)	0.1612 (3)	−0.06175 (8)	0.0153 (3)	
H3	0.4451	0.2703	−0.1031	0.018*	
C4	0.39762 (12)	0.0159 (3)	0.13132 (8)	0.0144 (3)	
C5	0.41723 (12)	−0.1539 (3)	0.19221 (8)	0.0163 (3)	
H5	0.4667	−0.2856	0.1962	0.020*	
C6	0.35315 (13)	−0.1034 (3)	0.24751 (9)	0.0190 (3)	
H6	0.3560	−0.1990	0.2916	0.023*	
C7	0.28728 (13)	0.0999 (3)	0.22898 (9)	0.0205 (3)	
H7	0.2408	0.1595	0.2592	0.025*	
C8	0.30066 (12)	0.5135 (3)	−0.05794 (8)	0.0161 (3)	
H8A	0.2722	0.4229	−0.1059	0.019*	
H8B	0.3624	0.6160	−0.0643	0.019*	
C9	0.20894 (12)	0.6715 (3)	−0.03966 (8)	0.0162 (3)	
H9A	0.2394	0.7674	0.0069	0.019*	
H9B	0.1504	0.5663	−0.0294	0.019*	
C10	0.15873 (13)	0.8453 (3)	−0.10717 (9)	0.0173 (3)	
H10A	0.2181	0.9460	−0.1184	0.021*	
H10B	0.1269	0.7482	−0.1532	0.021*	
C11	0.06820 (13)	1.0129 (3)	−0.09008 (9)	0.0180 (3)	
H11A	0.1007	1.1141	−0.0451	0.022*	
H11B	0.0102	0.9122	−0.0770	0.022*	
C12	0.01499 (13)	1.1804 (3)	−0.15839 (9)	0.0206 (3)	
H12A	0.0732	1.2796	−0.1718	0.025*	
H12B	−0.0182	1.0791	−0.2032	0.025*	
C13	−0.07480 (13)	1.3507 (3)	−0.14123 (10)	0.0237 (4)	
H13A	−0.1337	1.2538	−0.1291	0.036*	
H13B	−0.1050	1.4511	−0.1861	0.036*	
H13C	−0.0422	1.4544	−0.0977	0.036*	

### Atomic displacement parameters (Å^2^)

**Table:**

	*U*^11^	*U*^22^	*U*^33^	*U*^12^	*U*^13^	*U*^23^
S1	0.0231 (3)	0.0238 (3)	0.0156 (2)	0.00695 (14)	0.00873 (17)	0.00228 (13)
O1	0.0210 (6)	0.0222 (6)	0.0139 (5)	0.0084 (4)	0.0074 (4)	0.0046 (4)
C1	0.0131 (7)	0.0172 (7)	0.0143 (7)	0.0013 (6)	0.0021 (5)	−0.0007 (6)
C2	0.0135 (7)	0.0185 (7)	0.0117 (7)	−0.0007 (6)	0.0028 (5)	−0.0012 (5)
C3	0.0166 (7)	0.0174 (7)	0.0115 (7)	0.0015 (6)	0.0022 (5)	0.0024 (5)
C4	0.0126 (7)	0.0175 (7)	0.0124 (7)	0.0002 (5)	0.0016 (5)	−0.0023 (5)
C5	0.0158 (7)	0.0220 (8)	0.0119 (7)	−0.0011 (6)	0.0049 (6)	−0.0027 (6)
C6	0.0197 (7)	0.0247 (8)	0.0128 (7)	−0.0018 (6)	0.0042 (6)	0.0007 (6)
C7	0.0211 (8)	0.0286 (8)	0.0138 (7)	0.0016 (6)	0.0079 (6)	−0.0008 (6)
C8	0.0175 (7)	0.0189 (7)	0.0116 (7)	0.0033 (6)	0.0030 (5)	0.0018 (5)
C9	0.0165 (7)	0.0184 (7)	0.0139 (7)	0.0018 (6)	0.0040 (6)	−0.0001 (6)
C10	0.0184 (7)	0.0188 (7)	0.0146 (7)	0.0028 (6)	0.0039 (6)	0.0008 (6)
C11	0.0188 (7)	0.0181 (7)	0.0170 (7)	0.0019 (6)	0.0041 (6)	−0.0003 (6)
C12	0.0193 (8)	0.0235 (8)	0.0193 (8)	0.0048 (6)	0.0054 (6)	0.0026 (6)
C13	0.0207 (8)	0.0236 (8)	0.0257 (8)	0.0060 (6)	0.0037 (6)	0.0011 (7)

### Geometric parameters (Å, º)

**Table:**

S1—C7	1.7071 (15)	C8—H8A	0.9700
S1—C4	1.7380 (15)	C8—H8B	0.9700
O1—C1	1.3697 (18)	C9—C10	1.530 (2)
O1—C8	1.4326 (17)	C9—H9A	0.9700
C1—C3	1.388 (2)	C9—H9B	0.9700
C1—C2	1.409 (2)	C10—C11	1.524 (2)
C2—C3^i^	1.404 (2)	C10—H10A	0.9700
C2—C4	1.471 (2)	C10—H10B	0.9700
C3—C2^i^	1.404 (2)	C11—C12	1.527 (2)
C3—H3	0.9300	C11—H11A	0.9700
C4—C5	1.394 (2)	C11—H11B	0.9700
C5—C6	1.423 (2)	C12—C13	1.526 (2)
C5—H5	0.9300	C12—H12A	0.9700
C6—C7	1.362 (2)	C12—H12B	0.9700
C6—H6	0.9300	C13—H13A	0.9600
C7—H7	0.9300	C13—H13B	0.9600
C8—C9	1.513 (2)	C13—H13C	0.9600
			
C7—S1—C4	92.24 (7)	C8—C9—H9A	109.4
C1—O1—C8	118.42 (11)	C10—C9—H9A	109.4
O1—C1—C3	123.50 (14)	C8—C9—H9B	109.4
O1—C1—C2	115.58 (13)	C10—C9—H9B	109.4
C3—C1—C2	120.92 (14)	H9A—C9—H9B	108.0
C3^i^—C2—C1	117.07 (13)	C11—C10—C9	112.96 (12)
C3^i^—C2—C4	119.49 (13)	C11—C10—H10A	109.0
C1—C2—C4	123.42 (13)	C9—C10—H10A	109.0
C1—C3—C2^i^	122.00 (14)	C11—C10—H10B	109.0
C1—C3—H3	119.0	C9—C10—H10B	109.0
C2^i^—C3—H3	119.0	H10A—C10—H10B	107.8
C5—C4—C2	126.00 (13)	C10—C11—C12	113.12 (13)
C5—C4—S1	110.17 (11)	C10—C11—H11A	109.0
C2—C4—S1	123.82 (11)	C12—C11—H11A	109.0
C4—C5—C6	112.47 (14)	C10—C11—H11B	109.0
C4—C5—H5	123.8	C12—C11—H11B	109.0
C6—C5—H5	123.8	H11A—C11—H11B	107.8
C7—C6—C5	112.76 (14)	C13—C12—C11	113.32 (13)
C7—C6—H6	123.6	C13—C12—H12A	108.9
C5—C6—H6	123.6	C11—C12—H12A	108.9
C6—C7—S1	112.35 (12)	C13—C12—H12B	108.9
C6—C7—H7	123.8	C11—C12—H12B	108.9
S1—C7—H7	123.8	H12A—C12—H12B	107.7
O1—C8—C9	107.75 (11)	C12—C13—H13A	109.5
O1—C8—H8A	110.2	C12—C13—H13B	109.5
C9—C8—H8A	110.2	H13A—C13—H13B	109.5
O1—C8—H8B	110.2	C12—C13—H13C	109.5
C9—C8—H8B	110.2	H13A—C13—H13C	109.5
H8A—C8—H8B	108.5	H13B—C13—H13C	109.5
C8—C9—C10	111.36 (12)		

Symmetry code: (i) −*x*+1, −*y*, −*z*.

### Hydrogen-bond geometry (Å, º)

Cg1 is the centroid of the S1, C4–C7 ring.

**Table:**

*D*—H···*A*	*D*—H	H···*A*	*D*···*A*	*D*—H···*A*
C5—H5···*Cg*1^ii^	0.93	2.85	3.5809 (16)	137

Symmetry code: (ii) −*x*+1, *y*−1/2, −*z*+1/2.
